# An Overview of Systematic Reviews of Chinese Herbal Medicine for Alzheimer’s Disease

**DOI:** 10.3389/fphar.2021.761661

**Published:** 2021-11-26

**Authors:** Nanyang Liu, Tingting Zhang, Jiahui Sun, Jiuxiu Yao, Lina Ma, Jianhua Fu, Hao Li

**Affiliations:** ^1^ Xiyuan Hospital, China Academy of Chinese Medical Sciences, Beijing, China; ^2^ College of First Clinical Medicine, Shandong University of Traditional Chinese Medicine, Jinan, China; ^3^ Graduate School, Beijing University of Chinese Medicine, Beijing, China; ^4^ Wangjing Hospital, China Academy of Chinese Medical Sciences, Beijing, China

**Keywords:** Chinese herbal medicine, traditional Chinese medicine, Alzheimer’s disease, overview, systematic review and meta-analysis

## Abstract

**Background:** Multiple systematic reviews (SRs) have been conducted to evaluate the efficacy and safety of Chinese herbal medicine (CHM) in patients with Alzheimer’s disease (AD). Here, we aim to perform an overview to assess the methodological quality and quality of evidence of the SRs to provide convincing data on the treatment of AD with CHM.

**Method:** Six electronic databases including Chinese and English were searched, until April 31, 2021. Two researchers independently screen documents and extract data according to the predesigned rules. A Measure Tool to Assessment System Reviews 2 (AMSTAR-2) was used to investigate the methodological quality, and the Grading of Recommendations, Assessment, Development, and Evaluation (GRADE) was used to determine the quality of evidence for outcomes.

**Results:** Twelve qualified SRs including 163 randomized controlled trials were reviewed. The methodological quality of the included SRs was considered extremely low assessed through AMSTAR-2. Compared with western medicines (WM) alone, CHM as an adjuvant treatment has shown significant effects in improving Mini-mental State Examination, Alzheimer’s Disease Assessment Scale-Cognitive, and Clinical Dementia Rating scores. The same is true for CHM alone. Regarding the effect on Activities Daily Living, neither the single CHM nor the combination with WM has an obvious effect. For the total effective rate, both single CHM and the combination with WM shown significant effects. Nine SRs suggested that CHM as adjuvant therapy or single-use had fewer adverse events than WM. Additionally, the quality of evidence for the main outcome was reviewed as low or extremely low according to GRADE profiler data.

**Conclusion:** Current evidence suggests that CHM may be beneficial in improving the cognitive function of AD patients. However, we should be cautious about the evidence due to methodological flaws and low quality. High-quality RCTs are further needed to confirm the efficacy and safety of CHM for AD.

## 1 Introduction

Alzheimer’s disease (AD), a progressively worsening neurodegenerative disease, is the most common type of dementia that threatens the health of the elderly. As of 2019, more than 50 million people worldwide suffer from dementia, and this number will increase to 152 million by 2050 (ADI., 2019). Data from the Alzheimer’s disease survey in the United States show that between 2000 and 2018, the number of deaths caused by stroke, AIDS, and heart disease gradually decreased, while deaths caused by AD increased by 146.2% (Alzheimer’s Association., 2020). As global aging intensifies, finding effective treatments to prevent disease progression presents a major challenge.

As for the pathogenesis of AD, most of the published reviews have been focused on either the role of neuroinflammation, oxidative stress, synaptic abnormalities, and neuronal cell apoptosis (Sung et al., 2020). However, despite the above knowledge, the underlying mechanism is still unclear. At present, several approved drugs can only improve the symptoms, but cannot prevent the conversion of mild cognitive impairment to dementia (Long and Holtzman, 2019). While some of the latest potential drug developments have partially shed some light on this issue, it continues to be difficult to find available alternatives (Daly et al., 2020).

It is noteworthy that, traditional Chinese medicine (TCM) has a long history in the healthcare of AD patients in China (Pei et al., 2020). Chinese herbal medicine (CHM) as the main pharmacological treatment of TCM has been extensively studied in recent years because of the potential therapeutic benefits. In this context, there is pioneering evidence supporting the occurrence of dynamic interplays between herbal extracts and their biological complexes and AD through anti-oxidant, anti-apoptotic and anti-inflammatory, that, contribute to partially improve the cognitive decline (Pei et al., 2020). In the past few years, many systematic reviews (SRs) have been conducted to evaluate the potential therapeutic benefits of CHM for AD patients (Zeng et al., 2015; Ma et al., 2018; Zhang et al., 2019). However, the conclusions are inconsistent due to the quality of the primary research and method defects.

Overview of systematic reviews is a novel tool used to solve specific and focused issues related to policies and practices (Onasanya et al., 2016). The purpose is to synthesize the evidence from multiple SRs into one available document, which can be used to guide healthcare professionals and decision-makers (Bero et al., 1998; Lewin et al., 2008). Herein, we conducted a qualitative review to critically evaluate the methodological quality and the quality of evidence of numerous SRs, and then comprehensively evaluate the evidence of CHM that can be used for AD.

## 2 Methods

### 2.1 Search Strategy

Two researchers independently searched the PubMed, Embase, Cochrane Library, China Biomedical Literature Database, China National Knowledge Infrastructure, Wan Fang Database of China, until April 30, 2021. The language of publication is limited to Chinese and English. The search terms were as follows: Chinese medicine, Traditional Chinese Medicine, Herbal medicine, Chinese herbal medicine, traditional medicine, CHM, Alzheimer’s disease, dementia, and meta-analysis. For the Chinese database, the above search terms use Chinese accordingly.

### 2.2 Eligibility Criteria

Qualified studies meet the following criteria: 1) Study: a systematic review of randomized controlled trials reports the effects of CHM on AD; 2) Participant: subjects identified as AD according to diagnostic criteria. There are no restrictions on gender, age, race, duration, and disease intensity; 3) Intervention: the treatment group adopts CHM or combined with WM, regardless of the drug form, dosage, frequency, and duration; 4) Comparison: the comparison group adopts WM or placebo; 5) Outcome: cognitive function assessment, including a series of cognitive scales, such as Mini-mental State Examination (MMSE), Alzheimer’s Disease Assessment Scale-Cognitive section (ADAS-Cog), Montreal Cognitive Assessment (MoCA), Hasegawa Dementia Scale (HDS), Clinical Dementia Rating (CDR), and Activities Daily Living (ADL). The incidence of adverse events and the total effective rate were also included.

Studies that meet the following qualifications were excluded: 1) network meta-analysis, SR without meta-analysis, review articles, editorials, conference abstracts, case reports, and repeated studies; 2) documents for which complete data are not available; 3) the control group uses any one or more than two CHM therapy.

### 2.3 Study Selection and Data Collection

Retrieved records were imported into the document management system. Initial screening was performed by reading the title and abstract after removing duplicates. The second screening is based on reading the full text, and the controversial literature is determined through discussion between two researchers or consultation for the third researcher. To ensure data integrity and consistency, the two researchers used pre-designed data extraction tables to extract data. We obtain the following data: general information (first author, journal, publication year, country/region, funding source), participant characteristics (age, gender, race, education level, disease stage, and severity), research characteristics (sample size, study design, follow-up time), intervention measures, and outcomes.

### 2.4 Assessing the Quality of SR

The methodological quality of the SR was assessed through AMSTAR-2 (Shea et al., 2007). It is a tool for evaluating methodological quality consisting of 16 items, including seven key items (2, 4, 7, 9, 11, 13, and 15), each item can be evaluated as Yes, Partial Yes, or No. According to the number of violations of key items, the quality of research is divided into four levels: high, medium, low, or extremely low.

### 2.5 Assessing the Quality of Evidence

The quality of the evidence for the outcome is determined by GRADE’s four levels (high, medium, low, or extremely low) (Guyatt et al., 2008). When faced with the risk of bias, inconsistency, imprecision, indirectness, or publication bias, the evidence is reduced. Conversely, when faced with large effect sizes, dose-response, and adjustments for confounding factors, the evidence is improved. GRADE profiler 3.6 software is used to assess the level of evidence.

### 2.6 Data Synthesis

We provide a narrative description of the included SRs. Tabulate all the primary and secondary outcomes, and extract the pooled effect size. The risk ratio (RR) and 95% confidence interval (CI) were used to summarize the dichotomous variables, and the weighted mean difference (WMD) or standard mean deviation (SMD) and 95% CI were used to summarize the continuous data. Obtain the heterogeneity of each included SR, which is detected by the *I* ^2^ and Chi^2^ tests.

## 3 Results

**FIGURE 1:**
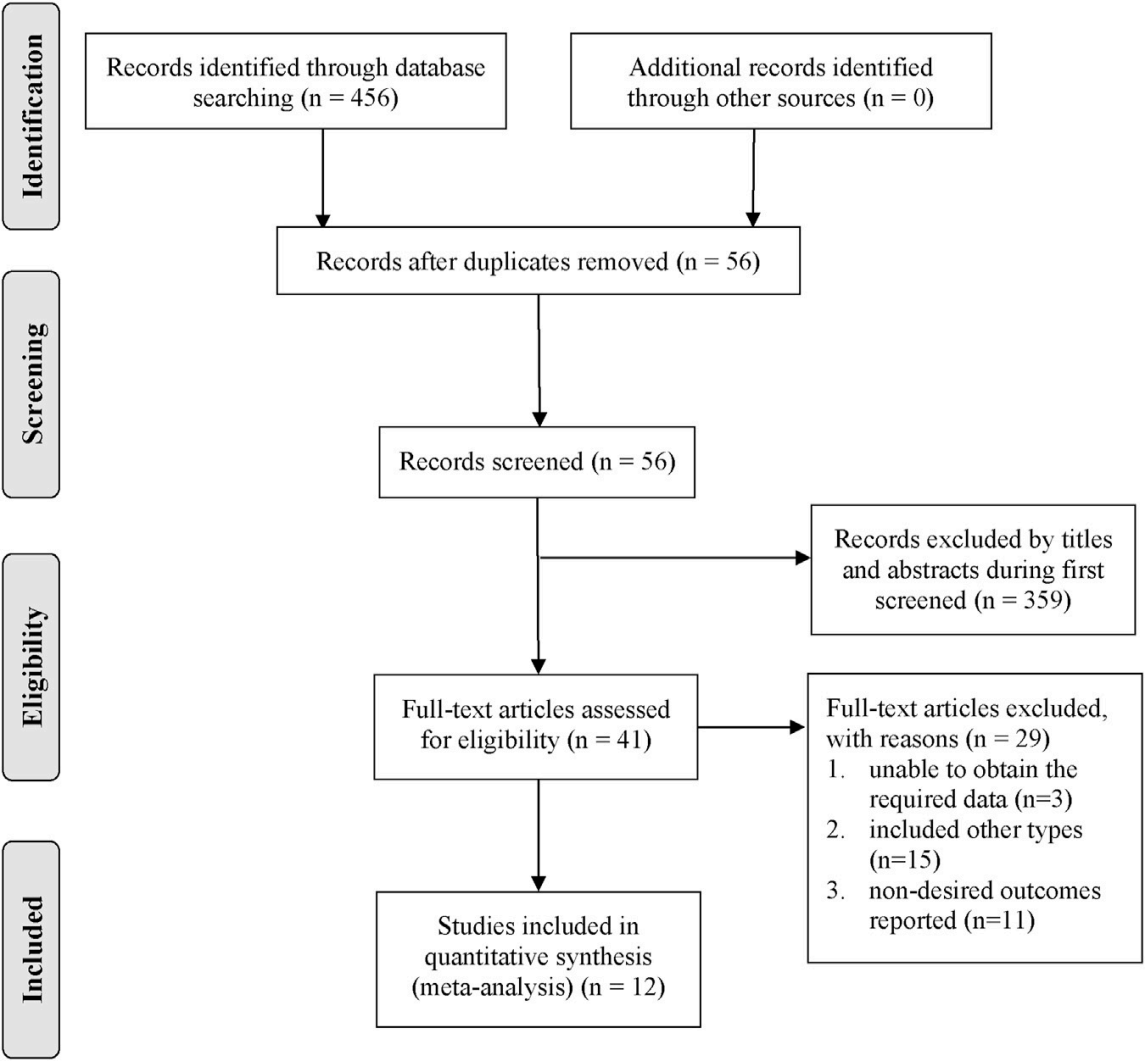
Summarizes the detailed search process.

A total of 456 records were initially retrieved from six databases. All records were imported into the document manager, and 56 duplicate records were screened out. Through reading the titles and abstracts, 359 records irrelevant to the subject were excluded, and the remaining 41 were read in full. We excluded 29 articles that did not meet the inclusion criteria through reading the full text, and finally obtained 12 articles for review (Xu and Xie, 2015; Zeng et al., 2015; Du et al., 2017; Ma et al., 2018; Chen et al., 2019; Li et al., 2019; Qin et al., 2019; Shi et al., 2019; Yang et al., 2019; Zhang et al., 2019; Wang et al., 2020; Yang et al., 2021). Figure 1 summarizes the detailed search process.

### 3.1 Study Characteristics

The 12 included SRs were published between 2014 and 2020. The number of original studies in these SRs ranged from 5 to 39, with samples ranging from 213 to 3440. Three SRs used the Jadad scale to assess the quality (Xu and Xie, 2015; Ma et al., 2018; Yang et al., 2019), and the rest used the bias risk assessment tool recommended by Cochrane Library (Zeng et al., 2015; Du et al., 2017; Chen et al., 2019; Li et al., 2019; Qin et al., 2019; Shi et al., 2019; Zhang et al., 2019; Wang et al., 2020; Yang et al., 2021). All SRs mainly involve 12 outcomes, including the Activities Daily Living (ADL), Mini-mental State Examination (MMSE), Alzheimer’s Disease Assessment Scale-Cognitive section (ADAS-Cog), TCM syndrome curative effect, TCM syndrome integral, adverse events, total effective rate, etc. The basic characteristics of the included literature are shown in Supplementary Table S1.

### 3.2 AMSTAR-2 Method Evaluation Results

The methodological quality of all studies were evaluated as extremely low. Among the seven key items, none of the studies mentioned research protocols and guiding documents, six studies were not systematically searched (Zeng et al., 2015; Ma et al., 2018; Chen et al., 2019; Yang et al., 2019; Zhang et al., 2019; Wang et al., 2020), none of the studies provided a document exclusion list and related reasons for exclusion, seven studies did not use appropriate statistical methods combined effect estimates (Xu and Xie, 2015; Chen et al., 2019; Li et al., 2019; Shi et al., 2019; Yang et al., 2019; Zhang et al., 2019; Yang et al., 2021), and nine studies did not explain the risk of bias(Xu and Xie, 2015; Zeng et al., 2015; Ma et al., 2018; Chen et al., 2019; Li et al., 2019; Shi et al., 2019; Yang et al., 2019; Zhang et al., 2019; Wang et al., 2020; Yang et al., 2021). The details of the assessment of the quality of SRs are listed in Table 1.

**TABLE 1:** Methodological quality of included systematic reviews using AMSTAR-2.

References	Item 1	Item 2	Item 3	Item 4	Item 5	Item 6	Item 7	Item 8	Item 9	Item 10	Item 11	Item 12	Item 13	Item 14	Item 15	Item 16	Overall quality
Yang et al.	Y	N	Y	PY	Y	N	N	PY	Y	Y	N	N	Y	N	Y	N	Extremely low
Wang et al.	N	N	Y	N	Y	Y	N	N	Y	Y	Y	N	Y	N	Y	N	Extremely low
Li et al.	Y	N	Y	PY	N	Y	N	PY	Y	Y	N	N	Y	N	N	N	Extremely low
Qin et al.	Y	N	Y	PY	Y	Y	N	PY	Y	Y	Y	Y	Y	N	N	N	Extremely low
Yang et al.	Y	N	Y	N	N	N	N	PY	PY	Y	N	N	Y	Y	Y	N	Extremely low
Shi et al.	Y	N	Y	PY	N	N	N	N	Y	Y	N	N	N	N	N	N	Extremely low
Chen et al.	Y	N	Y	N	Y	Y	N	PY	PY	Y	N	Y	Y	N	Y	N	Extremely low
Zhang et al.	Y	N	Y	N	N	Y	N	PY	Y	Y	N	N	N	N	Y	Y	Extremely low
Du et al.	Y	N	Y	PY	N	Y	N	PY	Y	Y	Y	Y	Y	Y	Y	N	Extremely low
Ma et al.	Y	N	Y	N	N	Y	N	PY	PY	Y	Y	N	Y	Y	N	Y	Extremely low
Xu et al.	Y	N	Y	PY	N	N	N	PY	Y	Y	N	N	Y	N	Y	N	Extremely low
Zeng et al.	Y	N	Y	N	Y	Y	N	PY	Y	Y	Y	N	Y	Y	Y	Y	Extremely low

Y, yes; N, no; PY, partial yes.

### 3.3 Effects of the CHM Intervention

#### 3.3.1 Cognitive Performance

##### 3.3.1.1 Chinese Herbal Medicine Vs. Western Medicines

The effectiveness of CHM on MMSE was evaluated in 6 SRs (Ma et al., 2018; Li et al., 2019; Shi et al., 2019; Zhang et al., 2019; Wang et al., 2020; Yang et al., 2021), four of which showed that CHM is better than WM in improving cognitive function (Shi et al., 2019; Zhang et al., 2019; Wang et al., 2020; Yang et al., 2021), while two reported no statistical difference (Ma et al., 2018; Li et al., 2019). Two study suggested that there was no significant difference between CHM and WM in improving ADAS-Cog (Li et al., 2019; Yang et al., 2021). One SR suggested that CHM is better than WM in improving CDR scores(Ma et al., 2018).

##### 3.3.1.2 Chinese Herbal Medicine Combined Western Medicines Vs. Western Medicines

Six SRs evaluated the potential benefits of CHM combined with WM on cognitive function, five were for MMSE, and one were for ADAS-Cog. Among the 6 SRs, CHM combined WM improved cognitive function better than WM alone, while there was no significant statistical difference in one study (Yang et al., 2019).

#### 3.3.2 Activities Daily Living

##### 3.3.2.1 Chinese Herbal Medicine Vs. Western Medicines

The potential efficacy of CHM on ADL was evaluated in six SRs, however, inconsistent results were found. Two studies showed that CHM was inferior to WM in improving ADL (Shi et al., 2019; Yang et al., 2021), one was the opposite (Zhang et al., 2019), and three had no statistical difference (Ma et al., 2018; Li et al., 2019; Wang et al., 2020). Four SRs investigated the effect of CHM combined with WM on ADL. Two studies found that CHM combined with WM improved ADL inferiorly to WM alone (Du et al., 2017; Qin et al., 2019), one was the opposite (Chen et al., 2019), and the other had no statistical difference (Yang et al., 2019).

#### 3.3.3 Total Effective Rate

Eight studies reported the total effective rate of CHM alone or in combination with WM on AD (Xu and Xie, 2015; Zeng et al., 2015; Chen et al., 2019; Li et al., 2019; Qin et al., 2019; Shi et al., 2019; Yang et al., 2019; Wang et al., 2020), including 7,768 participants in 110 RCT trials. Except for one SR, the total effective rate of CHM or in combination with WM is better than WM alone (Zeng et al., 2015).

#### 3.3.4 Adverse Events

Nine SRs out of 12 studies evaluated adverse events. Although all studies claim that there is no significant difference in adverse events or side effects between CHM and WM, only one SR has quantified data. Three SRs even show that compared with WM, AD patients treated with CHM have a significantly lower incidence of certain symptoms such as dizziness, tinnitus, headache, and angina (Zeng et al., 2015; Du et al., 2017; Ma et al., 2018). However, these results were only concentrated in a small number of trials. The safety assessment of a larger sample size is further needed.

#### 3.3.5 Summary of Quality of Evidence

Although we did not reassess the risk of bias in the RCTs contained in SRs, it is necessary to review the results reported by the authors to assess the quality of these trials to use GRADE to determine the overall quality of the evidence. As mentioned in these reports, many of the reviewed trials are of poor quality and have a high risk of bias, which leads to a decline in the quality of evidence. Although the beneficial effects of CHM on each outcome are frequently reported, these findings are usually based on highly heterogeneous. Among all the degrading factors, the risk of bias in the original trial is the most prominent, followed by publication bias, inconsistency, and imprecision. All studies found no factors for an upgrade. Overall, the quality of the evidence for each result reported was very low. The details of quality of evidence in included SRs were generalized in Table 2.

**TABLE 2:** Quality of evidence in the included systematic reviews based on GRADE.

References	Outcomes	No.of	Risk of bias	Inconsistency	Indirectness	Imprecision	Publication bias	No. of participants		Pooled effect size	95% CI	Quality of evidence
								Intervention	Control			
Yang et al. (2021)	ADL	9	−1^a^	0	0	0	−1^b^	283	279	MD = −1.30	(-2.27, -0.33)	Low
	MMSE	10	−1^a^	−1^c^	0	0	−1^b^	317	313	MD = 1.17	(0.15, 2.20)	Extremely low
	ADAS-Cog	4	−1^a^	−2^d^	0	−1^e^	−1^b^	123	121	MD = −4.69	(-16.14, 6.75)	Extremely low
	TCM-SSS	4	−1^a^	−2^d^	0	−1^e^	−1^b^	108	104	MD = −4.70	(-8.97, -0.43)	Extremely low
	AR	7	−1^a^	NR	0	−1^e^	NR	NR	NR	NR	NR	Not evaluated
Wang et al. (2020)	ADL	35	−2^f^	−2^d^	0	−1^e^	−1^b^	1,006	901	MD = −1.00	(-4.56, 2.56)	Extremely low
	MMSE	29	−2^f^	−2^d^	0	0	−1^b^	1,348	1,229	MD = 1.82	(0.91, 2.72)	Extremely low
	AR	8	−2^f^	NA	0	0	−1^b^	NR	NR	NR	NR	Not evaluated
	TER	39	−2^f^	0	0	0	−1^b^	1784	1,656	RR = 1.14	(1.03, 1.25)	Extremely low
Li et al. (2019)	ADL	5	−1^a^	−1^c^	0	−1^e^	−1^b^	187	169	SMD = 0.27	(-0.41, 0.95)	Not evaluated
	MMSE	8	−2^f^	−2^d^	0	0	−1^b^	247	221	WMD = 1.87	(0.76, 2.98)	Extremely low
	AR	1	−2^f^	NA	0	NR	NR	24	22	NR	NR	Not evaluated
	TER	6	−2^f^	0	0	0	−1^b^	222	212	RR = 1.29	(1.16, 1.44)	Extremely low
Qin et al. (2019)	ADL	6	−1^a^	−2^d^	0	−1^e^	−1^b^	224	180	WMD = −0.52	(-0.76, -0.28)	Extremely low
	MMSE	8	−1^a^	−1^c^	0	0	−1^b^	320	280	WMD = 0.48	(0.31, 0.64)	Extremely low
	ADAS-Cog	3	−1^a^	−1^c^	0	−1^e^	−1^b^	92	90	WMD = −0.41	(-0.71, -0.11)	Extremely low
	AR	5	−1^a^	−1^c^	0	NR	NR	192	150	NR	NR	Not evaluated
	TER	9	−1^a^	0	0	0	−1^b^	371	330	OR = 3.37	(2.35, 4.83)	Low
Yang et al. (2019)	ADL	5	−2^f^	−1^c^	0	−1^e^	−1^b^	165	161	WMD = 0.81	(-2.46, 0.83)	Extremely low
	MMSE	6	−2^f^	0	0	0	−1^b^	247	241	WMD = 1.34	(0.74, 1.95)	Extremely low
	AR	7	−2^f^	−1^c^	0	−1^e^	NR	NR	NR	NR	NR	Not evaluated
	TRE	7	−2^f^	0	0	0	−1^b^	205	201	OR = 2.28	(1.47, 3.52)	Extremely low
Shi et al. (2019)	ADL	4	−2^f^	−1^c^	0	0	−1^b^	283	277	MD = −3.40	(-4.92, -1.87)	Extremely low
	MMSE	5	−2^f^	−2^d^	0	0	−1^b^	314	308	MD = 2.69	(2.13, 3.26)	Extremely low
	AR		−2^f^	−1^c^	0	−1^e^	−1^b^	NR	NR	NR	NR	Not evaluated
	TER	3	−2^f^	0	0	−1^e^	−1^b^	77	67	RR = 1.27	(1.08, 1.50)	Extremely low
Chen et al. (2019)	ADL	3	−2^f^	−1^c^	0	−1^e^	−1^b^	149	152	MD = 10.48	(8.33, 12.64)	Extremely low
	AR	5	−2^f^	0	0	0	−1^b^	234	237	OR = 0.31	(0.19, 0.49)	Extremely low
	TER	9	−2^f^	0	0	0	−1^b^	412	415	OR = 2.07	(1.39, 3.07)	Extremely low
Zhang et al. (2019)	ADL	16	−1^a^	0	0	0	0	NR	NR	SMD = 0.38	(0.25, 0.49)	Moderate
	MMSE	23	−1^a^	−1^c^	0	0	0	NR	NR	SMD = 0.66	(0.44, 0.89)	Low
Du et al. (2017)	ADL	5	−2^f^	0	0	−1^e^	−1^b^	160	156	MD = −3.60	(-4.53, -2.66)	Extremely low
	MMSE	7	−2^f^	−1^c^	0	0	−1^b^	238	238	MD = 2.69	(1.46, 3.92)	Extremely low
	ADAS-Cog	3	−2^f^	−1^c^	0	−1^e^	−1^b^	88	83	MD = −4.54	(-5.64, -3.43)	Extremely low
Ma et al. (2018)	CDR	1	−1^a^	−1^c^	0	−1^e^	−1^b^	31	15	MD = −0.40	(-0.69, -0.11)	Extremely low
	ADL	NA	−1^a^	−1^c^	0	−1^e^	−1^b^	NR	NR	MD = 0.94	(-1.54, 3.43)	Extremely low
	MMSE	5	−1^a^	0	0	−1^e^	−1^b^	114	109	MD = 0.69	(-0.17, 1.56)	Extremely low
	AR	NA	−1^a^	−1^c^	0	−1^e^	−1^b^	NR	NR	NR	NR	Not evaluated
Xu and Xie, (2015)	MMSE	4	−2^f^	−1^c^	0	−1^e^	−1^b^	125	126	MD = 2.87	(0.64, 5.10)	Extremely low
	TER	7	−2^f^	0	0	0	−1^b^	278	246	OR = 1.25	(1.14, 1.38)	Extremely low
Zeng et al. (2015)	MMSE	15	−1^a^	−1^c^	0	−1^e^	−1^b^	717	575	MD = 0.79	(-0.11, 1.69)	Extremely low
	AR		−1^a^	−1^c^	0	−1^e^	−1^b^	NR	NR	NR	NR	Not evaluated
	TER	15	−1^a^	0	0	0	−1^b^	717	575	OR = 1.09	(0.82, 1.46)	Low

NR, Not reported; TCM-SSS, Traditional Chinese Medicine syndrome score scale; ADL, Activies Daily Living; MMSE, Mini-mental State Examination; ADAS-Cog, Alzheimer’s Disease Assessment Scale-Cognitive section; AR, adverse reactions; TER, total effective rate; SMD, standard mean difference; MD, mean difference; OR, odds ratio.

a The included studies have a high risk of bias in terms of randomization, blinding, allocation concealment, completeness of result data, or selective reporting.

b Asymmetric funnel plot or less than nine studies.

c 75% ≤ *I* ^2^ ≤ 100%.

d 50% ≤ *I* ^2^ < 75%.

e small sample studies accounted for the majority.

f The included studies have two or more high risks of bias in terms of randomization, blinding, allocation concealment, completeness of result data, or selective reporting.

## 4 Discussion

In recent years, multiple SRs have been conducted to clarify the potential efficacy and safety of CHM for AD. However, the results are inconsistent due to differences in methods and regions. Therefore, we conducted this review to synthesize multiple SRs and evaluate their methodological quality and level of evidence.

This is the first overview of systematic reviews to comprehensively evaluate the efficacy and safety of CHM in the treatment of AD. We identified 12 SRs with meta-analysis in this field, covering 59 RCTs. Overall, the AMSTAR-2 assessment is good or acceptable for most SRs. Based on the existing evidence, our review confirmed that CHM has a potentially beneficial effect on improving cognitive function and overall clinical impression and that it has a better effect when used in combination with WM. Our review also showed that there is insufficient evidence to support the beneficial effects of CHM on the activities of daily living. Additionally, none of the included SRs reported any adverse events in patients receiving Chinese herbal medicine, which seems to be safe for AD patients.

However, we found that all included SRs were considered extremely low quality. Specifically, in key item two of AMSTAR-2, only one study performed the registration and wrote the study protocol, and only one study was completed under the guidance of the PRISMA statement, which increased the risk of bias and affected the rigor of SRs. In key item 4, most SRs only provide search keywords and databases, but no specific search strategies and restrictions, which makes it difficult to ensure the comprehensiveness and repeatability of the literature search. In key item seven, all included SRs did not provide a list of exclusion documents and reasons for exclusion, which may undermine transparency and affect the reliability of the results. In key item 11, most studies did not use appropriate statistical methods to test for heterogeneity, nor did they give reasons to explain the source of heterogeneity. In addition, the included SRs have defects in the risk of bias, heterogeneity, publication bias, and financial support information, which affects the effectiveness of the quality of evidence. Moreover, it was found that the results reported in these studies were of low level of evidence-based on GRADE evaluation.

Although the overview of systematic review provides a broad perspective on interventions and their relative effectiveness, it inevitably has some limitations. Firstly, although our search strategy seems thorough, we cannot completely rule out the possibility that related SRs published in other languages will be missed. Secondly, major trials recently published may be omitted from the included SRs. However, the search date of the included comment is the most recent. Thirdly, the evaluation of SRs rather than the original RCT may not be able to obtain relevant details of the main study. The included SRs were performed on low-quality raw data, so the results are extremely susceptible to bias, which is a major limitation of this overview. Nevertheless, in this review, the quality of the evidence has been verified.

## 5 Conclusion

Current evidence suggests that CHM may be beneficial in improving the cognitive function of AD patients. However, due to the methodological limitations of the primary trial and the low quality of the evidence, the results should be interpreted and summarized carefully. A further rigorously designed RCT is needed to evaluate the benefits of CHM in the treatment of dementia, which should be large-scale and multi-center and should follow the relevant guidelines and procedures for the biological treatment of AD. Moreover, the safety of CHM for AD patient needs further evaluation. However, it should be remembered that lack of scientific evidence does not necessarily mean ineffective (Kotsirilos, 2005). Therefore, CHM treatment for AD patients should be open. Alzheimer’s Disease International, 2019.

## Data Availability

The raw data supporting the conclusion of this article will be made available by the authors, without undue reservation.
