# Supplementary material for: An Overview of Systematic Reviews of Chinese Herbal Medicine for Alzheimer’s Disease
Source: Front Pharmacol. 2021 Nov 26;12:761661. doi: 10.3389/fphar.2021.761661 (PMC8664324; doi:10.3389/fphar.2021.761661)
Supplement: Supplementary file 1 [file Table1.DOCX]

| **Supplementary table 1 Study characteristics of included systematic reviews** | | | | | | | | | |  |
| --- | --- | --- | --- | --- | --- | --- | --- | --- | --- | --- |
| **References** | **Year** | **Study**  **design** | **No.of**  **study** | **No. of**  **patient** | **Intervention** | **Control** | **Quality**  **assessment tool** | **Outcomes** | **Primary conclusion** | |
| Yang et al | 2021 | RCT | 14 | 846 | kidney-tonifying Decoction (*Panax ginseng* C.A.Mey., *Epimedium brevicornum* Maxim., *Morinda officinalis* How. ) | Donepezil | Cochrane  bias risk | ①②③  ④⑤ | CHM therapy is beneficial to improve MMSE score and CHM syndrome score, and its efficacy is better than donepezil | |
| Wang et al | 2020 | RCT | 39 | 3440 | Huanglian Jiedu Decoction  (*Coptis chinensis* Franch., *Scutellaria baicalensis* Georgi, *Phellodendron chinense* Schneid., *Gardenia jasminoides* Ellis) | Donepezil | Cochrane  bias risk | ①②⑤⑥ | The total effective rate of CHM in the treatment of AD is better than that of WM. CHM can improve patients' MMSE and ADL scores | |
| Shi et al | 2019 | RCT | 6 | 672 | Huanglian Jiedu Decoction  (*Coptis chinensis* Franch., *Scutellaria baicalensis* Georgi, *Phellodendron chinense* Schneid., *Gardenia jasminoides* Ellis) | Donepezil | Cochrane  bias risk | ①②⑤⑥ | CHM is safe and effective in treating AD, which can improve cognitive function and ability daily life | |
| Li et al | 2019 | RCT | 8 | 559 | kidney-tonifying Decoction (*Panax ginseng* C.A.Mey., *Epimedium brevicornum* Maxim., *Morinda officinalis* How. ) | Donepezil | Cochrane  bias risk | ①②③④  ⑤⑥ | CHM treatment of AD is better than WM in total clinical efficacy, and has low adverse reactions | |
| Ma et al | 2016 | RCT | 6 | 596 | Ginkgobiloba | Donepezil | Jadad | ①②⑤ | Compared with donepezil, there is no significant difference in the efficacy of oral Chinese medicine in AD patients | |
| Zhang et al | 2019 | RCT | 25 | 1855 | Ginkgobiloba | Donepezil | Cochrane  bias risk | ①② | Compared with WM, CHM has the potential to improve the cognitive function and ADL of AD patients. | |
| Qin et al | 2019 | RCT | 10 | 764 | Yiqi Congming Decoction combined Donepezil (*Astragalus membranaceus* Bge, *Glycyrrhiza uralensis*, *Panax ginseng* C.A.Mey., *Cimicifuga heracleifolia* Kom., *Pueraria lobata* Ohwi, *Vitex trifolia* L., *Paeonia latiflora* Pall., *Phellodendron chinense* Schneid. ) | Donepezil | Cochrane  bias risk | ①②③⑤  ⑥ | CHM can improve the clinical efficacy of senile dementia, improve cognitive function and self-care ability in daily life, and have fewer adverse reactions | |
| Yang et al | 2019 | RCT | 7 | 488 | Bushen Huoxue Decoction  combined Donepezil (*Epimedium brevicornum* Maxim., *Morinda officinalis* How., *Paeonia suffruticosa* Andr., *Panaxnotoginseng* F.H.Chen) | Donepezil | Jadad | ①②⑤⑥ | The efficacy of CHM in the treatment of AD is significantly better than that of Western medicine alone, without serious adverse reactions | |
| Xu et al | 2015 | RCT | 11 | 780 | kidney-tonifying Decoction combined Donepezil (*Panax ginseng* C.A.Mey., *Epimedium brevicornum* Maxim., *Morinda officinalis* How. ) | Donepezil | Jadad | ②⑥ | CHM combined with WM is indeed effective in treating AD | |
| Du et al | 2017 | RCT | 8 | 605 | Ginkgobiloba,  or combined Donepezil | Donepezil | Cochrane  bias risk | ①②③ | The clinical efficacy of CHM combined with donepezil in the treatment of AD is better than donepezil alone, with high safety | |
| Chen et al | 2019 | RCT | 9 | 827 | Liuwei Dihuang Decoction combined Donepezil (*Rehmannia glutinosa* Libosch, *Dioscorea opposite Thunb*., *Cornus officinalis* Sieb. et Zucc., *Wolfiporia* cocos, *Alisma plantago-aquatica* Linn., *Paeonia suffruticosa* Andr.) | Donepezil | Cochrane  bias risk | ①⑤⑥ | CHM treatment of AD is better than WM in total clinical efficacy, and has fewer adverse reactions | |
| Zeng et al | 2015 | RCT | 20 | 1682 | Ginkgobiloba,  or combined WM | Donepezil | Cochrane  bias risk | ②⑤⑥ | Unable to determine the effectiveness and safety of oral Chinese medicine for AD | |
| ①:ADL②:MMSE③:ADAS-cog④:CHM Syndrome Score Scale ⑤:Adverse reactions⑥:Total effective rate  RCT: randomized controlled trial, CHM: Chinese Herbal Medicine, WM: western medicine, AD: Alzheimer's disease, ADL: Activies Daily Living, MMSE: Mini-mental State Examination , ADAS-Cog: Alzheimer’s Disease Assessment Scale-Cognitive section. | | | | | | | | | |  |
